# Understanding Neuronal Architecture in Obesity through Analysis of White Matter Connection Strength

**DOI:** 10.3389/fnhum.2016.00271

**Published:** 2016-06-06

**Authors:** Justin W. Riederer, Megan E. Shott, Marisa Deguzman, Tamara L. Pryor, Guido K. W. Frank

**Affiliations:** ^1^Department of Psychiatry, University of Colorado School of Medicine, University of Colorado Anschutz Medical CampusAurora, CO, USA; ^2^Neuroscience Program, University of Colorado Denver, Anschutz Medical CampusAurora, CO, USA; ^3^Eating Disorders Center DenverDenver, CO, USA

**Keywords:** obesity, white matter, tractography, connectivity, connection strength

## Abstract

Despite the prevalence of obesity, our understanding of its neurobiological underpinnings is insufficient. Diffusion weighted imaging and calculation of white matter connection strength are methods to describe the architecture of anatomical white matter tracts. This study is aimed to characterize white matter architecture within taste-reward circuitry in a population of obese individuals. Obese (*n* = 18, age = 28.7 ± 8.3 years) and healthy control (*n* = 24, age = 27.4 ± 6.3 years) women underwent diffusion weighted imaging. Using probabilistic fiber tractography (FSL PROBTRACKX2 toolbox) we calculated connection strength within 138 anatomical white matter tracts. Obese women (OB) displayed lower and greater connectivity within taste-reward circuitry compared to controls (Wilks’ *λ* < 0.001; *p* < 0.001). Connectivity was lower in white matter tracts connecting insula, amygdala, prefrontal cortex (PFC), orbitofrontal cortex (OFC) and striatum. Connectivity was greater between the amygdala and anterior cingulate cortex (ACC). This study indicates that lower white matter connectivity within white matter tracts of insula-fronto-striatal taste-reward circuitry are associated with obesity as well as greater connectivity within white matter tracts connecting the amygdala and ACC. The specificity of regions suggests sensory integration and reward processing are key associations that are altered in and might contribute to obesity.

## Introduction

Worldwide, obesity is recognized as the fifth leading cause of death (World Health Organization, 2009). Genetic, physiological, lifestyle, community-based and additional factors all contribute to the development of the obese phenotype (Pate et al., 2013). Thus, obesity is an extremely complex disease to study and effective, specific interventions remain elusive. Research aimed at uncovering the neurobiological mechanisms that underlie obesity can advance our understanding of obesity, develop a more thorough understanding of risk factors and will inform clinical intervention for the disease.

A wide range of homeostatic, cortical and limbic structures have been associated with the neurobiology of obesity (Berthoud and Morrison, 2008). More specifically, altered taste and taste-reward circuitry may contribute to obesity as it governs motivation to eat and mediates food intake (Morton et al., 2006; Berridge et al., 2010). This underlying circuitry begins in the mouth with its taste receptors and continues in neurons projecting from the brainstem to the insula and frontal operculum, and is subsequently integrated with our sense of sight and smell through subcortical, cortical and limbic structures (Rolls, 2015). Understanding this neurobiology *in vivo* is key to understanding the development and maintenance of obesity. Investigation of these mechanisms has been undertaken using an array of magnetic resonance imaging (MRI) paradigms.

Previous functional MRI (fMRI) studies in obesity have shown *hyper*-active response to visual, taste and olfactory food cues in various brain regions as well as *hypo*-active brain response in the striatum and dorsolateral prefrontal cortex (PFC), which are associated with reward processing and executive control (Carnell et al., 2012). fMRI research that specifically examines taste processing has also shown that obesity is associated with attenuated brain response in the striatum, insula and PFC (Frank et al., 2012). These functional findings are promising but only answer a portion of the question pertaining to the neurobiological mechanisms at play in obesity. Clear description of the structural neurobiology which may contribute to obesity is needed to provide a complete picture.

Diffusion weighted imaging can further describe the structural neurobiology of obesity. A recent review of diffusion weighted imaging in obesity identified 19 studies assessing fractional anisotropy (FA), radial diffusivity (RD) and medial diffusivity (MD) in obese individuals (Kullmann et al., 2015). By measuring the diffusion of water molecules within the brain, FA, RD and MD can be calculated, which allows for examination of axonal microstructure and can provide information on axonal ordering, axonal density and degree of myelination (Jones et al., 2013). In regard to these measures of axonal microstructure, the review illustrated that increased body mass index (BMI) was consistently correlated with lower FA in the corpus callosum, fornix, cingulum and corona radiata. The review also reported on just five studies examining anatomical white matter connectivity through tractography (Kullmann et al., 2015).

Tractography analysis allows for computational reconstruction of anatomical white matter tracts (Jones et al., 2013). This technique has been utilized in obesity research to show shorter anatomical white matter connections in the temporal lobe and fewer white matter fibers in the midcingulate cortex among populations of obese individuals (Kullmann et al., 2015). Tractography analysis is needed in order to uncover these anomalous white matter connectivity patterns, and despite having the potential to further clarify and describe the underlying neurobiology of obesity, tractography has been underutilized in obesity research.

Since publication of the aforementioned review, two additional groups have used deterministic tractogrophy to report on white matter connectivity in overweight and obese populations. The first study used a classification algorithm to show that overweight and lean individuals can be accurately classified based on signatures in anatomical white matter tracts connecting reward, salience, executive control and emotional arousal networks (Gupta et al., 2015). The authors also illustrated that overweight status is associated with increased fiber density between reward network regions and regions of the executive control, emotional arousal and somatosensory networks. Decreased fiber density between the ventromedial PFC and the anterior insula as well as between the thalamus and regions of the executive control network was also reported (Gupta et al., 2015). The second study assessed white matter connectivity in a limited reward network and found fewer reconstructed white matter fibers between reward network regions including the orbitofrontal cortex (OFC), caudate, putamen and accumbens as well as lower fiber integrity within the same tracts in obese individuals (Marqués-Iturria et al., 2015). Given the limited nature of tractography analyses in obesity, further investigation is warranted and has the potential to clarify the role anatomical white matter connectivity patterns may play in the disease.

The current study is aimed to characterize anatomical correlates that may contribute to the pathophysiology of obesity. We sought to understand whether there were white matter fiber distribution differences within the taste-reward circuitry between healthy and obese individuals using probabilistic tractography. Given evidence suggesting that obesity is associated with chronic low-grade inflammation (Aguilar-Valles et al., 2015) and the association between neuroinflammations and neurodegeneration (Glass et al., 2010), we hypothesized that connectivity within anatomical white matter tracts connecting taste-reward regions would be lower in obese individuals.

## Materials and Methods

### Subjects

This study was carried out in accordance with the recommendations of the Colorado Multiple Institutional Review Board with written informed consent from all subjects. All subjects gave written informed consent in accordance with the Declaration of Helsinki. We examined obese (*n* = 18, age = 28.7 ± 8.3 years) and healthy control (*n* = 24, age = 27.4 ± 6.3 years) women. Control women (CW) had a lifetime history of healthy weight (18.5 ≤ BMI ≤ 25 kg/m^2^), had no clinical eating or weight concerns and were free from lifetime major medical or psychiatric illness. Obese women (OB) had a BMI ≥ 30 kg/m^2^ and were free from major medical illness. Individuals taking medication other than oral contraceptive were excluded from the study.

### Behavioral Measures

All subjects completed behavioral measures of eating disorder psychopathology, state and trait anxiety and sensitivity to reward and punishment. Eating disorder psychopathology was assessed using the Eating Disorder Inventory (EDI; Garner, 2004). Anxiety scores were based on the State/Trait Anxiety Inventory for Adults (STAI Y-1; Spielberger, 1983). Sensitivity to reward and punishment were assessed using the Sensitivity to Punishment and Sensitivity to Reward Questionnaires (SPSRQ; Torrubia et al., 2001).

Subjects also completed a subjective taste test rating. Subjects tasted 2 ml of 1 molar sucrose solution (85.75 g sucrose in 250 ml H_2_O) and artificial saliva (0.928 g KCl + 0.084 g NaHCO_3_ in 500 ml H_2_O) and rated each solution for pleasantness and sweetness on a Likert scale. Taste testing was completed prior to image collection on the morning of the MRI.

### MRI Acquisition

Structural brain images were acquired on a GE Signa 3T scanner. Diffusion weighted images included 25 diffusion directions and one T2-weighted (*b* = 0) baseline image. Each image included 45 slices acquired in anterior-posterior commissure orientation (128 × 128 matrix, TR/TE = 16,000/82.6 ms, field of view = 26 cm, *b*-value = 1000, ASSET, slice thickness/gap = 2.6/0 mm).

### Diffusion Weighted Image Processing

Diffusion weighted images were processed using FSL’s Diffusion Toolbox 4.1.3 (FDT, Oxford Centre for Functional MRI of the Brain^1^). Images were corrected for eddy current distortions and head motion. Probabilistic fiber tractography was computed for each subject using PROBTRACKX2 to generate the most probable connectivity distribution between seed and ipsilateral target. Tractography parameters were: 5000 sample tracts per seed voxel, 0.2 curvature threshold, step length of 0.5 and a maximum number of steps 2000. Connectivity was assessed by computing connection strength which determines the mean probability of streamlines for each seed-target combination. The calculated connection strength value was divided by the total connection probability of seed regions then multiplied by the mean connection probability across seed and target regions and finally divided by the target volume of interest in order to normalize and rescale the results for size of seed and target regions (Eickhoff et al., 2010). We also corrected for physical path length (Eickhoff et al., 2010).

**Figure 1:**
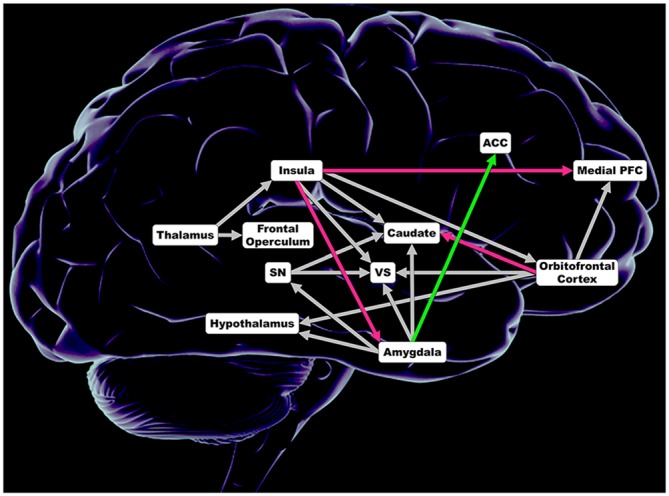
**Regions included in analysis of connection strength within 138 anatomical white matter tracts connecting regions comprising taste and taste-reward circuitry.** Magenta depicts lower connections strength in obese women (OB). Green illustrates greater connection strength in OB. Gray shows no significant difference in connection strength. ACC, anterior cingulate cortex; Insula = dorsal anterior insula, posterior insula and ventral anterior insula; Medial PFC, medial prefrontal cortex; Caudate = head of the caudate and caudate body; SN, substantia nigra; VS, ventral striatum; Orbitofrontal Cortex = inferior orbitofrontal cortex, medial orbitofrontal cortex, middle orbitofrontal cortex and gyrus rectus; Amygdala = basolateral amygdala and central nucleus of the amygdala.

In each hemisphere, tract based connection strength was calculated for anatomical white matter tracts connecting regions of a comprehensive taste-reward hierarchy proposed by Rolls (Figure 1; Rolls, 2011). Seed regions included the thalamus, dorsal anterior insula, ventral anterior insula, posterior insula, substantia nigra (SN), central nucleus of the amygdala, basolateral amygdala (BLA), medial OFC, middle OFC, gyrus rectus and inferior OFC. Thalamus targets included all subregions on the insula and the frontal operculum. Targets of insula subregions included the BLA, central nucleus of the amygdala, ventral striatum (VS), head of the caudate, body of the caudate, medial PFC, medial OFC, middle OFC, gyrus rectus and the inferior OFC. Targets of the SN were the VS, head of the caudate and body of the caudate. For both the central nucleus of the amygdala and the BLA, targets were the hypothalamus, SN, VS, head of the caudate, body of the caudate and the anterior cingulate cortex (ACC). OFC subregion targets included the hypothalamus, VS, head of the caudate, body of the caudate and the medial PFC. In total, tract based connection strength was calculated for 138 white matter tracts connecting aforementioned seed and ipsilateral targets. The Automated Anatomical Labeling atlas was used to determine coordinates for each seed and target region.

### Statistical Analyses

The extracted values for demographic variables, behavioral variables and tract based connection strength were submitted to multivariate ANOVAs (MANOVAs) in SPSS Statistics 22 (IBM Corp. Armonk, NY, USA) for analysis. The MANOVA model results were corrected for multiple comparisons (Bonferroni). False discovery rate correction was applied to correlation analysis of the relationship between demographic results, behavioral results and tract based connection strength.

## Results

### Demographic and Behavioral Results (Table 1)

Groups did not differ in terms of age. OB had significantly higher BMI compared to CW. OB scored significantly higher of several behavioral variables including drive for thinness, body dissatisfaction and sensitivity to punishment. Groups did not differ in perception of sweetness or pleasantness of 1 molar sucrose solution or artificial saliva.

**Table 1:** **Comparison of age, body mass index, behavioral results and sweetness and pleasantness perception between control and obese women**.

	Control women *n* = 24		Obese women *n* = 18			
	Mean	S.D.	Mean	S.D.	*t*	*p* value
Age (years)	27.42	6.28	28.67	8.30	−0.56	0.581
Body mass index (kg/m^2^)	21.64	1.26	34.78	4.44	−12.18	<0.001
Drive for thinness (EDI-3)	2.63	3.41	11.67	7.35	−4.84	<0.001
Body dissatisfaction (EDI-3)	4.38	4.25	26.17	9.04	−9.47	<0.001
Punishment sensitivity	4.04	1.85	6.78	4.29	−2.53	0.019
Reward sensitivity	4.42	2.84	6.22	4.49	−1.59	0.119
State anxiety	32.67	11.79	36.78	13.84	−1.04	0.305
Trait anxiety	33.92	11.35	39.44	11.16	−1.57	0.124
Pleasantness—Artificial saliva	2.96	2.60	2.11	2.11	1.13	0.265
Sweetness—Artificial saliva	1.25	0.74	1.17	0.38	0.44	0.665
Pleasantness—1 M sucrose	4.92	2.26	4.11	2.47	0.87	0.279
Sweetness—1 M sucrose	8.33	0.82	8.33	1.24	0.00	1.000

*S.D. = standard deviation*.

### Connectivity Results (Figure 1)

**Figure 2:**
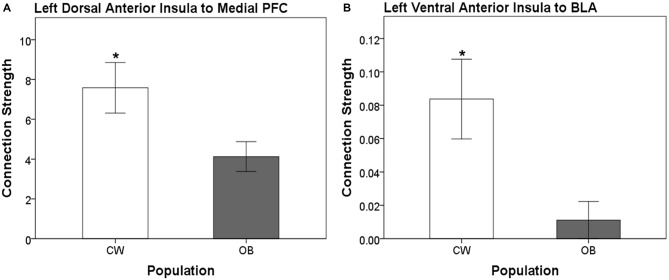
**(A)** Connection strength in white matter tracts connecting the left dorsal anterior insula and ipsilateral medial prefrontal cortex (PFC). **(B)** Connection strength in white matter tracts connecting the left ventral anterior insula and ipsilateral basolateral amygdala (BLA). Error bars depict the addition and subtraction of one standard error of the mean. CW, control women; OB, obese women; PFC, prefrontal cortex; BLA, basolateral amygdala; *p < 0.05.

**Figure 3:**
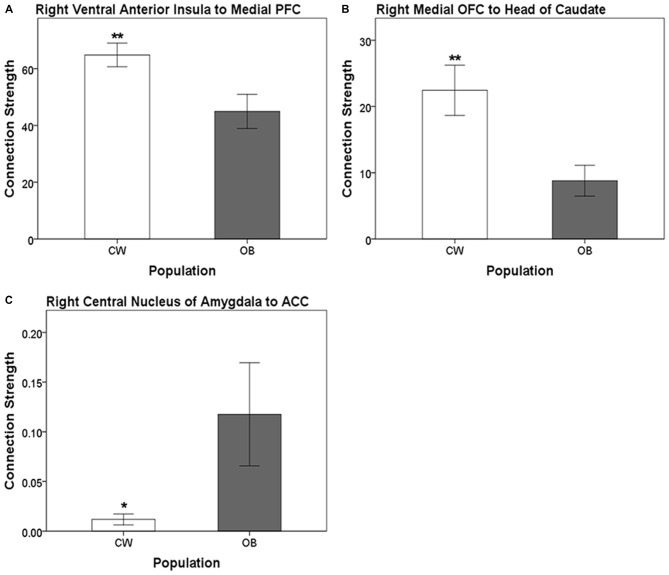
**(A)** Connection strength in white matter tracts connecting the right ventral anterior insula and ipsilateral medial PFC.** (B)** Connection strength in white matter tracts connecting the right medial OFC and ipsilateral head of the caudate. **(C)** Connection strength in white matter tracts connecting the right central nucleus of the amygdala and the ACC. Error bars depict the addition and subtraction of one standard error of the mean. CW, control women; OB, obese women; PFC, prefrontal cortex; OFC, orbitofrontal cortex; ACC, anterior cingulate cortex; *p < 0.05, **p < 0.01.

Distinct anatomical white matter connectivity was observed in OB (Wilks’ *λ* < 0.001; *p* < 0.001). Specifically, connectivity was lower in anatomical white matter tracts linking the left dorsal anterior insula to the left medial PFC and left ventral anterior insula to the left BLA (Figure 2). Additionally, OB had lower connectivity in white matter connections from the right ventral anterior insula to the right medial PFC and right medial OFC to the right head of the caudate (Figures 3A,B). Connectivity was greater in anatomical white matter tracts from the right central nucleus of the amygdala to the right ACC in OB (Figure 3C).

### Correlational results

Correlational analysis was performed to examine the relationship between white matter connectivity and behavioral scores of anxiety, eating disorder psychopathology and reward and punishment sensitivity. Correlations between connectivity and sweetness and pleasantness perception of 1 molar sucrose and artificial saliva were also examined. Finally, correlational analysis was performed to examine the relationship between BMI and connectivity. Significant correlations were observed; however, correlations did not persist after a false discovery rate correction was applied to the data.

## Discussion

To our knowledge, this study is the second to apply a probabilistic tractography analysis in a population of obese individuals (Kullmann et al., 2015). By applying a probabilistic method, which accounts for uncertainty in fiber direction at each voxel, this analysis is computationally more thorough than deterministic tractography methods (Descoteaux et al., 2009). The analysis further describes the role white matter connectivity may play in the neurobiology of obesity. OB have lower connectivity in anatomical white matter tracts between subregions of the left and right insula and medial PFC, left insula and amygdala and right OFC and head of the caudate compared to their control counterparts. However, greater connectivity between the right amygdala and ACC was observed in OB. Finding greater and lower anatomical white matter connectivity in OB suggest that specific anatomical alterations are associated with obesity. This is contrary to what we expected based on the FA literature, which overwhelmingly shows obesity being correlated with diminished axonal integrity (Stanek et al., 2011; Karlsson et al., 2013). Diffusion tensor imaging (DTI) methods that examine myelin more specifically also show a negative association between increased BMI and myelin properties (Kullmann et al., 2016). It is uncertain what the mechanism of increased and decreased white mater connection strength may be but obesity could affect white matter structure and food intake control (Hargrave et al., 2016). White matter tracts with lower connectivity between insula, PFC and OFC point to reward processing as well as sensory and cognitive integration as key processes that may be disrupted by the neuronal underpinnings of obesity. Greater connectivity provides additional evidence for specific white matter tracts disruptions in obesity and could be compensatory to lower connections (Wang et al., 2015).

White matter tracts with lower connectivity began at the insula; an area described as the primary taste cortex (Rolls, 2011). Specifically, we observed lower connectivity in white matter tracts originating in the dorsal anterior insula and ventral anterior insula. These subregions aid integration of cognitive and social-emotional stimuli respectively (Kurth et al., 2010). The anatomical white matter tracts where we observed lower connectivity terminated in the medial PFC and subregions of the amygdala. The medial PFC is important in integrating olfactory cues and subsequent decisions in response to olfactory cues (Rolls et al., 2010). Evidence from the animal literature has also shown that obese rats are more susceptible to olfactory food cues (Thanos et al., 2013), and while limited in human subject research, fMRI studies assessing response to olfactory cues show that obese individuals have increased activation in the hippocampus (Bragulat et al., 2010). Whether this finding suggests that obese individuals are more susceptible to olfactory food cues remains unclear. However, it is possible that obese individuals respond to olfactory cues in a manner that facilitates maladaptive decisions that perpetuate and exacerbate their physiological state. Furthermore, lower white matter connectivity could impact reward processing and the resulting motivation to eat. Taste activation in the insula is transmitted to the amygdala where experience and emotion are associated. The amygdala is also thought to drive dopamine activation in the reward cycle (Pauli et al., 2012). These findings suggest that sensory integration and reward processing may be key correlates of obesity. The role sensory integration and reward processing may play in obesity is strengthened by finding greater connectivity in additional white matter tracts.

Greater connectivity was found in white matter tracts connecting the amygdala and the ACC. Research in healthy adults has shown that pleasantness of oral fat texture is correlated with ACC activation, and the amygdala shows greater activation in response to high-fat taste (Grabenhorst et al., 2010). Additional research has shown that the amygdala and ACC activation is positively correlated with perception of increasing fat concentrations (Eldeghaidy et al., 2011). Resting-state functional connectivity analyses, which examine the statistical relationship between neuronal activation in spatially remote regions of the brain also show altered connectivity of the amygdala and ACC in obese populations (Lips et al., 2014; Wijngaarden et al., 2015). Our findings strengthen evidence suggesting that the ACC and amygdala are related to the neurobiology of obesity and may contribute to the obese phenotype via altered fat perception and processing. Greater connectivity also provides further evidence for altered reward processing in obesity as the ACC plays an important role in anticipation of food reward (Pelchat et al., 2004). Together these findings suggest that structural variations in taste-reward circuitry may contribute to obesity from initial presentation of taste stimuli to taste integration with other senses.

This study builds on previous analysis on white matter connectivity in taste-reward circuitry. Marqués-Iturria et al’s (2015) publication is, to our knowledge, the only previous study to examine reward network connectivity in obese individuals. Their analysis reported obesity being associated with lower connectivity of the bilateral caudate and nucleus accumbens to reward network regions (i.e., lateral OFC, medial OFC, caudate, putamen and accumbens). Our analysis also reports lower connectivity in white matter tracts projecting to the caudate, but also implicates additional white matter tracts as well as greater connectivity in specific anatomical connections. It is likely that differences in reported results are due to our analysis of a much larger and comprehensive taste-reward circuitry. The current study also adds to previous research from our group on gray and white matter volumes in OB. We previously showed that OB have diminished gray and white matter volumes in the amygdala, caudate, ACC, hippocampus, OFC and insula (Shott et al., 2015). These regions bear striking resemblance to the regions and associated white matter tracts implicated in this study. Further analysis of white matter connectivity that includes investigation of functional and effective connectivity within taste-reward circuitry may elucidate an interplay between anatomical connectivity and functional activation that could provide insight into possible mechanisms that alter gray and white matter volumes.

The specific mechanisms that led to these structural alterations also remain elusive based on this analysis. However, it is conceivable that neuroinflammation is a contributing factor to the observed differences in connectivity between OB and CW. It is understood that obesity is associated with a chronic low-grade inflammation, which is a risk factor for neuroinflammation (Aguilar-Valles et al., 2015). Many neurodegenerative diseases are also associated with neuroinflammation (Nguyen et al., 2014), and obesity has been correlated with cognitive decline and neurodegenerative diseases such as Alzheimer’s disease and dementia (Amor et al., 2010). Various studies have suggested that inflammation is associated with altered function in cognition and emotion processing circuits, which could affect normal eating (Castanon et al., 2015; Hargrave et al., 2016). The hypothesis of reduced fiber connectivity in response to inflammatory processes provides a plausible model, but the increase in fiber connections is more difficult to explain. However, it is possible that increases in fiber connectivity provide a compensatory process for fiber reduction, as seen in neurodegenerative processes (Wang et al., 2015). It is also plausible that differences in the dietary characteristics of OB and CW also impact white matter connectivity. The literature suggests that from birth through elderly age, dietary alterations can effect white matter connectivity and white matter volumes (de Kieviet et al., 2012; Deoni et al., 2013; Gu et al., 2015). This understanding is promising to our findings because it suggests that white matter structure alteration are fluid in nature and may be remedied by dietary changes. Alternatively, alteration may be trait-related to obesity. Further studies that examine those questions longitudinally are needed to clarify the interaction of these plausible mechanisms. Additionally, a better understanding of the behavioral implications of increased vs. decreased fiber connectivity is needed. Research has suggested that structural neurobiology may underpin behaviors associated with obesity (Iozzo, 2015). It is important that we continue research that examines structural neurobiology alongside behavior in order to further clarify how form may influence behavior.

### Limitations

Several aspects of this study may limit the findings. Only studying women limits the generalizability of this study. Also, probabilistic tractography measures the probability that connection strength or number of axons exceeds a certain value, but does not provide an absolute fiber count (Jones et al., 2013). However, probabilistic tractography does produce comparable results to white matter neuron tracing (Gao et al., 2013). Also, utilizing 25 diffusion directions during MRI data acquisition may limit probabilistic tractography analysis, however, it has been shown that increasing diffusion directions does not improve fiber-tracking (Berman et al., 2013). Lastly, we applied stringent statistical criteria to the data in order to reduce the probability of false discovery. While the stringent nature of our analysis is advantageous, it is possible that we eliminated significant results that would be realized with a larger sample size. Replication of these results in a large sample of men and women is needed to validate the results and further explore the relationship between white matter connectivity and the obese phenotype.

## Conclusion

This study shows that unique neurobiology underlies obesity. Connectivity within anatomical white matter tracts connecting insula-fronto-striatal and limbic regions may be neurobiological correlates of altered sensory integration and reward processing in obesity. Building this body of evidence has the potential to reduce stigma surrounding the disease. Further research is, however, necessary in order to progress the field. One specific question that the reported findings pose is how does neurobiological form influences function? In order to answer these questions, future studies of structural connectivity should conjointly examine the functional connectivity of regions through resting state MRI or effective connectivity analyses while performing obesity-related tasks, such as taste perception. Subsequent analyses also need to investigate if neurobiological abnormalities observed in obesity are state- or trait-related to the diseases. Examination of how structural neurobiology influences behavior also needs to continue. Clarifying these questions has the potential to improve and adapt current clinical interventions for the disease and improve care for those suffering from obesity. This will require large scale and longitudinal studies.

## Author Contributions

JWR, MES, MD, TLP and GKWF all meet and exceed the requirements for authorship and agree to be accountable for all aspects of the work.

## Funding

This work was supported by National Institute of Mental Health Grant No. R01 MH096777, NIMH Grant No. R01 MH103436 and by the Davis Foundation Award of the Klarman Family Foundation Grants Program in Eating Disorders (all GKWF).

## Conflict of Interest Statement

The authors declare that the research was conducted in the absence of any commercial or financial relationships that could be construed as a potential conflict of interest. The reviewer BW and handling Editor declared their shared affiliation, and the handling Editor states that the process nevertheless met the standards of a fair and objective review.

## Footnotes

^1^http://www.fmrib.ox.ac.uk/fsl
